# Trends and Disparities in Aortic Stenosis–Related Mortality Among Adults Aged ≥65 Years in the United States From 1999 to 2024

**DOI:** 10.1016/j.jscai.2026.105573

**Published:** 2026-08-11

**Authors:** Aymen Ahmed, Muteia Shakoor, Usama Hussain Kamal, Hiba Sohail, Muhammad Hasnain Mankani, Vikash Jaiswal, Najam Us Saqib, Nso Nso, Arman Qamar, Mark J. Ricciardi

**Affiliations:** a Section of Interventional Cardiology, Endeavor Center for Cardiovascular Intervention Outcomes Research and Evaluation (ECCORE), Endeavor Health Cardiovascular Institute, Glenview, Illinois; b The University of Chicago Pritzker School of Medicine, Chicago, Illinois; c Department of Medicine, Dow University of Health Sciences, Karachi, Pakistan; d Department of Medicine, Services Institute of Medical Sciences, Lahore, Pakistan; e Department of Medicine, Aga Khan University Hospital, Karachi, Pakistan

**Keywords:** aortic stenosis, epidemiology, health disparities, mortality trends, older adults, United States

## Abstract

**Background:**

Aortic stenosis (AS) remains a leading cause of cardiovascular death, yet temporal mortality trends specifically among older adults remain poorly characterized. This study analyzed trends and disparities in AS-related mortality among older adults in the United States from 1999 to 2024.

**Methods:**

Using the CDC WONDER database, we analyzed AS-related deaths among adults aged ≥65 years. Age-adjusted mortality rates (AAMR) and average annual percent change (AAPC) were calculated across demographic and geographic subgroups.

**Results:**

A total of 637,178 deaths were recorded. The overall AAMR for AS-related mortality decreased significantly from 1999 (55.74) to 2024 (52.46) (AAPC, –0.23; *P* =.004). Although rates rose from 2018 to 2021, a significant decline followed through 2024 (annual percent change, –2.40). Men had consistently higher AAMR than women; mortality remained stable in men (AAPC, –0.06; *P* = .33), but declined among women (AAPC, –0.35; *P* =.002). AS-related mortality decreased among patients aged 75 to 84 years, but increased in those ≥85 years (AAPC, 0.28), who had the highest AAMR throughout. Mortality declined in all census regions except for the Midwest, reaching the highest AAMR in 2024 (60.91). Nonmetropolitan areas saw increasing trends, whereas metropolitan areas saw declines. AAMR remained highest among non-Hispanic (NH) White individuals; however, significant decreases were observed in Hispanic/Latino and NH Asian/Pacific Islander populations.

**Conclusions:**

Aortic stenosis–related mortality declined modestly among older adults from 1999 to 2024. However, notable demographic and regional disparities persisted, including higher mortality in men, NH White individuals, older adults ≥85 years, and residents of nonmetropolitan regions and the Midwest. Further investigation and targeted interventions are needed to improve outcomes.

## Introduction

Aortic stenosis (AS) is the most prevalent valvular heart disease (VHD) in the United States (US), accounting for more than 60% of VHD-related mortality, predominantly affecting older adults.^1^^,^^2^ With the aging population, the burden of calcific aortic valve disease continues to grow, with an estimated 11.78 million older adults affected globally and projections suggesting a 72% increase in cases by 2041.^3^ Although many individuals with AS remain asymptomatic for prolonged periods, the onset of symptoms marks a critical turning point in disease progression. Once symptomatic, the prognosis is poor, with some studies suggesting that half of untreated patients die within 2 years, largely because of fluid overload, left ventricular decompensation, and heart failure.^4^

Historically, surgical aortic valve replacement (SAVR) has been the primary treatment for severe AS, improving both survival and quality of life.^4^ Over the past decade, the introduction of transcatheter aortic valve replacement (TAVR) has transformed the management of AS by providing a less invasive alternative to surgery. Although initially approved for patients at prohibitive or high surgical risk, TAVR indications have progressively expanded to include intermediate- and low-risk patients, substantially increasing treatment accessibility for older adults with AS. Despite these therapeutic advances, the population-level impact of evolving treatment strategies on AS-related mortality remains incompletely understood. Recent studies have evaluated mortality trends in VHD and AS in the US using national mortality data.^5^^,^^6^ However, most of these studies examined broader AS categories including both rheumatic and nonrheumatic etiologies, or evaluated mortality trends only through 2020, limiting insights into more recent patterns. Contemporary registry data including over 350,000 TAVR procedures from 2020 to 2023 highlight its widespread adoption in current practice, alongside national inpatient data highlighting an increase in TAVR utilization from 14,708 procedures in 2020 to 17,784 procedures in 2022.^7^^,^^8^ Given the increasing adoption of TAVR in recent years, persistent demographic and geographic disparities, and lack of studies evaluating nonrheumatic AS-related mortality specifically among older adults who bear the greatest mortality burden, we conducted this study to examine trends and disparities in nonrheumatic AS-related mortality among older adults in the US from 1999 to 2024 to provide insights into disease burden, treatment accessibility, and population-level trends.

## Materials and methods

### Data source

Mortality trends among adults with AS in the US were examined using data from the Centers for Disease Control and Prevention Wide-ranging Online Data for Epidemiologic Research (CDC WONDER) database.^9^ Records were identified using the International Classification of Diseases, Tenth Revision code I35.0 (nonrheumatic aortic [valve] stenosis). Other codes that may list AS secondarily were not included. Multiple-cause-of-death files and death certificate data were reviewed to determine whether AS was listed as an underlying or contributing cause of death. This study used publicly available, deidentified data from the CDC WONDER database. Because the data are publicly accessible and contain no personally identifiable information, institutional review board approval and informed consent were not required. The study was conducted in accordance with applicable ethical guidelines and regulations.

### Data extraction

Data for all 50 states and Washington, DC, in the United States were retrieved from the CDC WONDER database. The data set included demographic characteristics, population, year, and geographic areas. Demographic variables consisted of sex, age, race, and ethnicity. Sex was recorded as men or women on death certificates, and only individuals aged 65 years and older were included. Age was grouped into predefined categories: 65 to 69, 70 to 74, 75 to 79, 80 to 84, and 85+ years. Race and ethnicity classifications followed CDC WONDER conventions: non-Hispanic (NH) White, NH Black or African American, Hispanic or Latino, NH American Indian or Alaska Native, and NH Asian or Pacific Islander. Geographic data were organized using the National Center for Health Statistics Urban-Rural Classification Scheme for Counties, categorizing counties as large metropolitan (population ≥1 million), medium/small metropolitan (population 50,000-999,999), or rural (population <50,000). Broader US census regions were also assigned, including the Northeast, Midwest, South, and West.^10^ For all variables, data were extracted from years 1999 to 2024, except for urbanization and states, which were only available up to 2020 in the CDC WONDER database, and for racial subgroups, which were available through 2023.

### Statistical analysis

To assess AS-related mortality among adults aged ≥65 years, both crude and age-adjusted mortality rates (AAMR) per 100,000 individuals ≥65 years were computed. The crude mortality rate (CMR) was calculated by dividing the total number of AS-related deaths in a given year by the corresponding US population. AAMR rates were computed by standardizing AS mortality to the 2000 US standard population. Trends in age-adjusted mortality were assessed using the annual percent change (APC) and 95% CI, derived through the Joinpoint Regression Program (version 5.0, National Cancer Institute). Significant changes in AAMR for AS were identified by fitting log-linear regression models and evaluating temporal variations. Analyses for mortality trends in the overall population and age subgroups were carried out from 1999 through 2024, while those for the racial, urbanization, and state-level subgroups were carried out from 1999 through 2020 because of database limitations. An APC was classified as increasing or decreasing when the mortality trend’s slope differed significantly from 0, based on 2-tailed *t* tests. A *P* value <.05 was considered statistically significant. Subgroup analyses were prespecified and intended to descriptively evaluate temporal mortality patterns across demographic and geographic strata.

## Results

The Central Illustration highlights the key overall findings and demographic subgroup trends observed in our study.

**Central Illustration:**
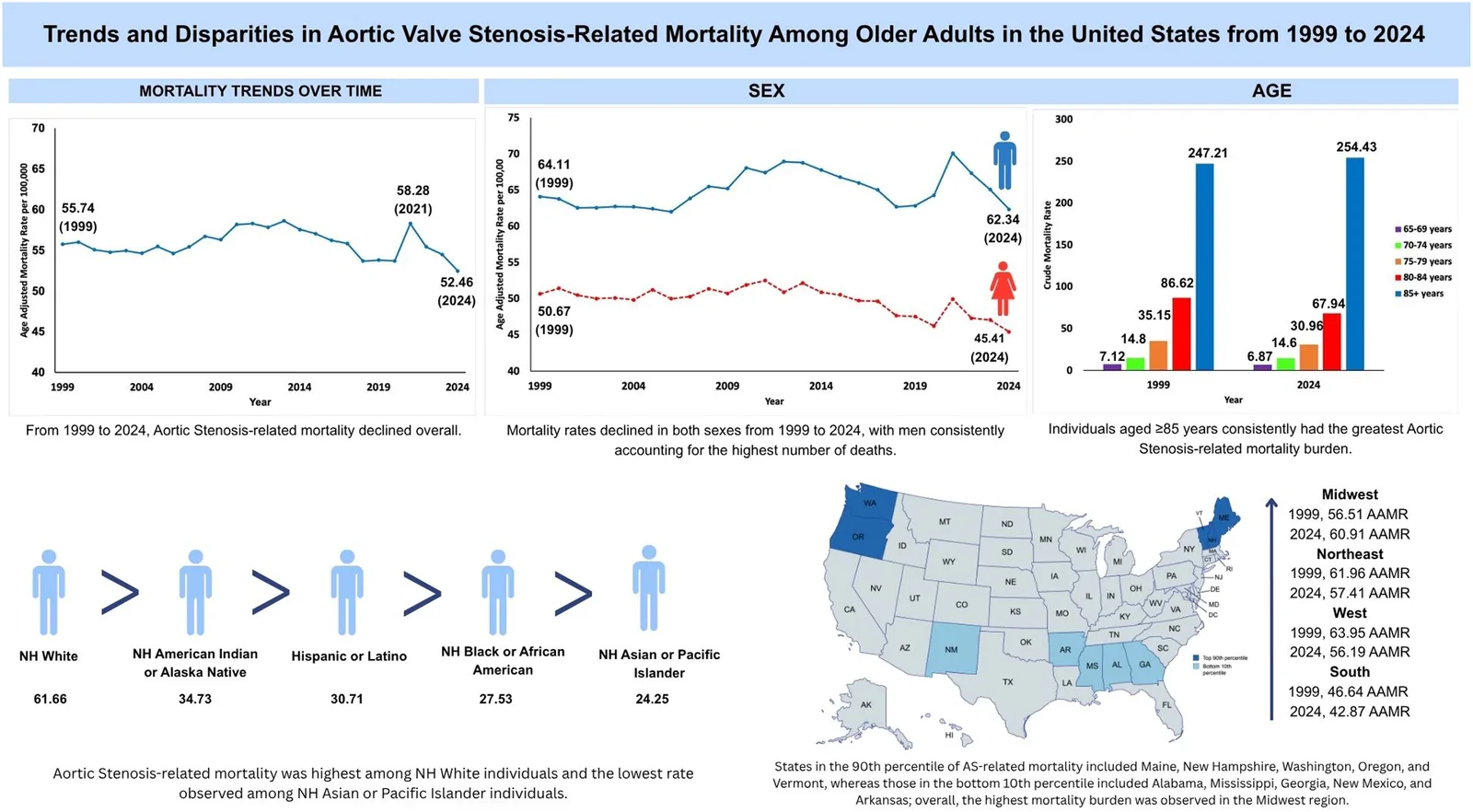
**Trends and disparities in aortic stenosis (AS)-related mortality among adults ≥65 years in the United States from 1999 to 2024**. AAMR, age-adjusted mortality rate; NH, non-Hispanic.

### Overall trend

From 1999 to 2024, 637,178 deaths were reported because of AS, representing roughly half of the 1,264,943 total deaths because of VHD. The AAMR for AS-related fatalities decreased from 55.74 in 1999 to 52.46 in 2024 (AAPC, –0.23; *P* = .004). From 2004 to 2013, there was a significant increase in AAMR with an APC of 0.88 (95% CI, 0.54-2.26; *P* = .008). This increase was followed by a significant decrease in AAMR with an associated APC of –1.85 (95% CI, –3.52 to –1.13; *P* = .008) from 2013 to 2018. Another significant increase in AAMR was seen from 2018 to 2021 with an associated APC of 1.88 (95% CI, 0.39-2.95; *P* = .01). A final significant decline was seen from 2021 to 2024 with an associated APC of –2.40 (95% CI, –4.67 to –1.33; *P* < .001) (Supplemental Table S1, Figure 1).

**Figure 1:**
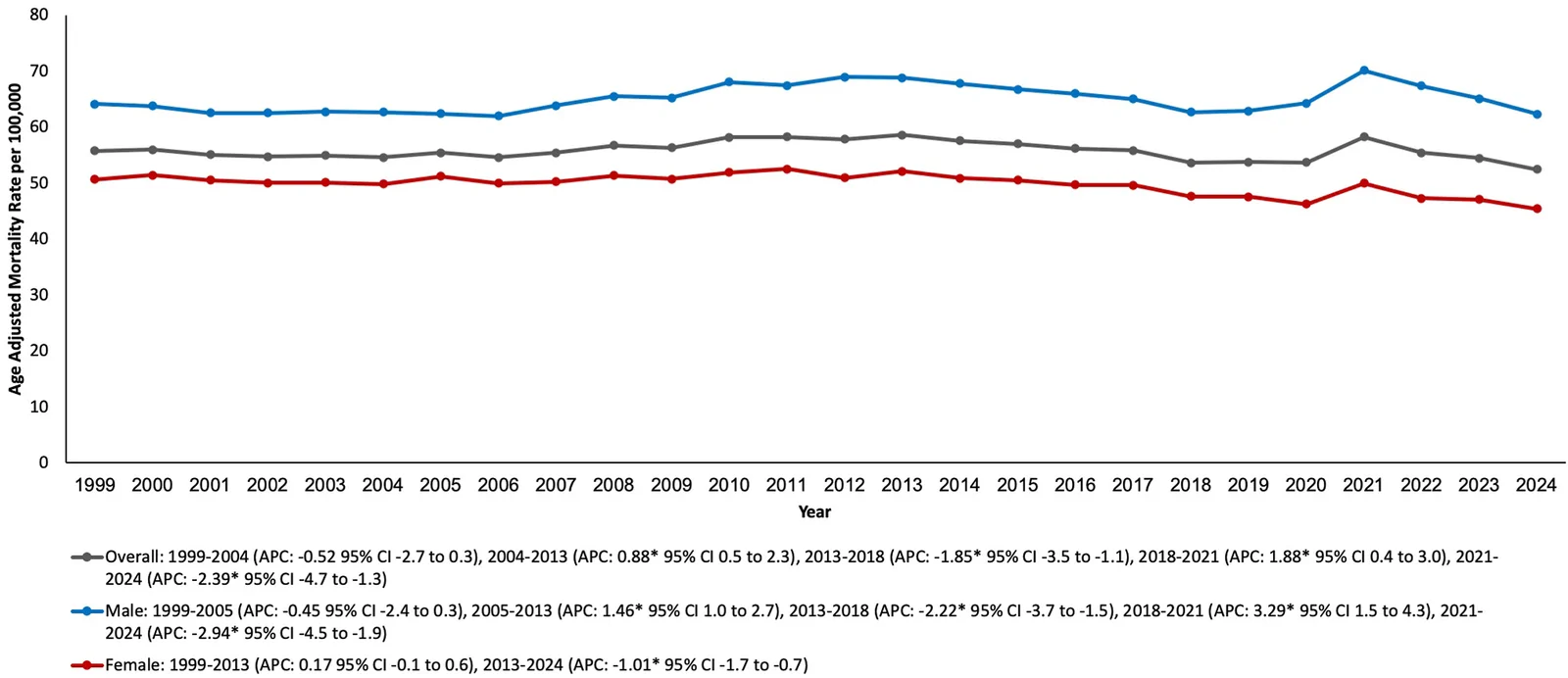
**Trends in overall and sex-stratified age-adjusted aortic stenosis–related mortality rates among adults in the United States, 1999 to 2024.** ∗Indicates that the annual percentage change (APC) significantly differs from 0 at α = 0.05.

### Mortality trends by demographic groups

#### Sex

Women exhibited a significant decline in mortality (AAPC, –0.35; *P* = .002), whereas men had a nonsignificant decrease (AAPC, –0.06; *P* = .33). Men were seen to have persistently higher AAMR than women throughout the study period. The AAMR for men was 64.11 in 1999 and 62.34 in 2024, while for women it was 50.67 in 1999 and 45.41 in 2024 (Supplemental Table S1, Figure 1). A nonsignificant increase in AAMR was observed in women from 1999 to 2013, with a subsequent significant decrease from 2013 to 2024, with an associated APC of 0.17 (95% CI, –0.10 to 0.63; *P* = .20) and –1.01 (95% CI, –1.67 to –0.66; *P* < .001), respectively. For men, a significant increase was observed from 2005 to 2013, and a significant decrease was observed from 2013 to 2018 with an associated APC of 1.46 (95% CI, 0.98-2.65; *P* < .001) and –2.22 (95% CI, –3.74 to –1.46; *P* < .001), respectively. This was followed by another significant rise in mortality from 2018 to 2021 with an associated APC of 3.29 (95% CI, 1.54-4.34; *P* < .001), and a subsequent significant decline from 2021 to 2024 with an associated APC of –2.94 (95% CI, –4.48 to –1.92; *P* < .0011).

#### Age

Throughout the study period, there was a consistent upward trend in CMR with increasing age. An overall decrease was observed in age groups 65 to 84 years, with a significant decline in the 75 to 79 (AAPC, –0.60; *P* < .001) and 80 to 84 years age group (AAPC, –0.94; *P* < .001). However, for individuals in the 85+ year age group, an overall significant increase in mortality was recorded with an AAPC of 0.28 (*P* = .003). The highest CMR were reported in the 85+ year age group with a significant increase from 1999 to 2012 with an APC of 1.13 (95% CI, 0.76-1.75; *P* < .001), followed by a significant decline from 2012 to 2024 with an APC of –0.63 (95% CI, –1.19 to –0.26; *P* < .001). For the 80 to 84 years age group, mortality decreased significantly from 1999 to 2005 (APC, –2.27; 95% CI, –3.26 to –1.57; *P* = .008), followed by a nonsignificant increase from 2005 to 2013 (APC, 0.95; 95% CI, –0.94 to 1.66; *P* = .07) and a subsequent decrease from 2013 to 2018 (APC, –3.08; 95% CI, –4.47 to 1.26; *P* = .12). Another nonsignificant increase was seen from 2018 to 2022 (APC, 0.90; 95% CI, –3.44 to 2.41; *P* = .16), followed by a period of significant decrease in mortality from 2022 to 2024 (APC, –2.67; 95% CI, –4.44 to –0.17; *P* = .02). Significant decline in mortality was seen for the 75 to 79 years age group from 1999 to 2006 (APC, –2.16; 95% CI, –3.98 to –1.47; *P* < .001), followed by a significant increase from 2006 to 2009 (APC, 2.85; 95% CI, 0.37-4.24; *P* = .01), and a significant decrease from 2009 to 2018 (APC, –1.21; 95% CI, –3.09 to –0.84; *P* < .001), respectively. This was followed by a significant rise in mortality from 2018 to 2021 (APC, 4.47; 95% CI, 1.91-5.84; *P* < .001) and a subsequent decrease from 2021 to 2024 (APC, –3.39; 95% CI, –5.74 to –2.03; *P* < .001). An initial significant decline, followed by a significant increase, was seen for the 70 to 74 years age group from 1999 to 2012 (APC, –0.93, 95% CI, –3.14 to –0.30; *P* = .005) and from 2012 to 2024 (APC, 0.96, 95% CI, 0.36-2.92; *P* = .003). A similar trend was observed for the 65 to 69 years age group from 1999 to 2005 (APC, –3.47; 95% CI, –8.00 to –1.47; *P* < .001) and 2005 to 2024 (APC, 0.92; 95% CI, 0.61-1.34; *P* < .001) (Supplemental Table S2, Figure 2).

**Figure 2:**
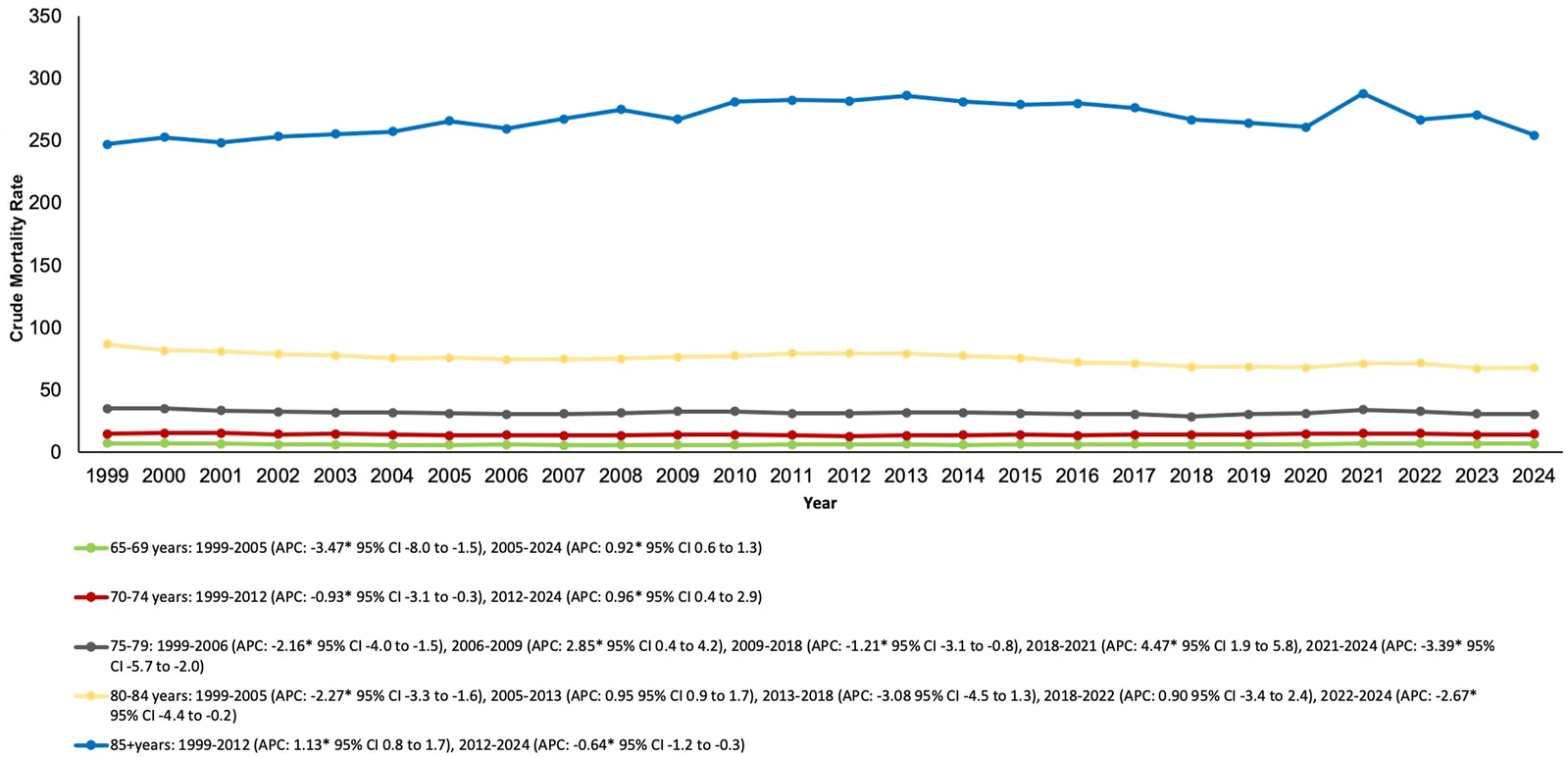
**Trends in aortic stenosis–related crude mortality rates stratified by age groups among adults in the United States, 1999 to 2024.** ∗Indicates that the annual percent change (APC) is significantly different than 0 at α = 0.05.

#### Race

When stratified by race or ethnicity, an overall decrease in AAMR was seen among NH American Indian or Alaska Native, Asian or Pacific Islander, and Hispanic or Latino. The highest overall AAMR was noted in NH White individuals (61.66), followed by NH American Indian or Alaska Native (34.73), Hispanic or Latino (30.71), NH Black or African American (27.53), and NH Asian or Pacific Islander (24.25). Over the study period, a significant decrease in mortality was observed for the Hispanic or Latino individuals (AAPC, –0.44; *P* = .007) and the NH Asian or Pacific Islanders (AAPC, –1.00; *P* < .001). A decrease in mortality was also seen in the NH American Indian or Alaska Native (AAPC, –0.04; *P* = .92); however, it was nonsignificant. An overall increase in mortality was seen among NH White (AAPC, 0.051; *P* = .71) and NH Black (AAPC, 0.05; *P* = .71), which was also nonsignificant. For NH White individuals, a significant increase in AAMR were seen from 2004 to 2013, followed by a significant decline from 2013 to 2020 with an associated APC of 1.13 (95% CI, 0.74-1.51; *P* < .001), and –1.04 (95% CI, –1.47 to –0.61; *P* < .001), respectively (Supplemental Table S3, Figure 3).

**Figure 3:**
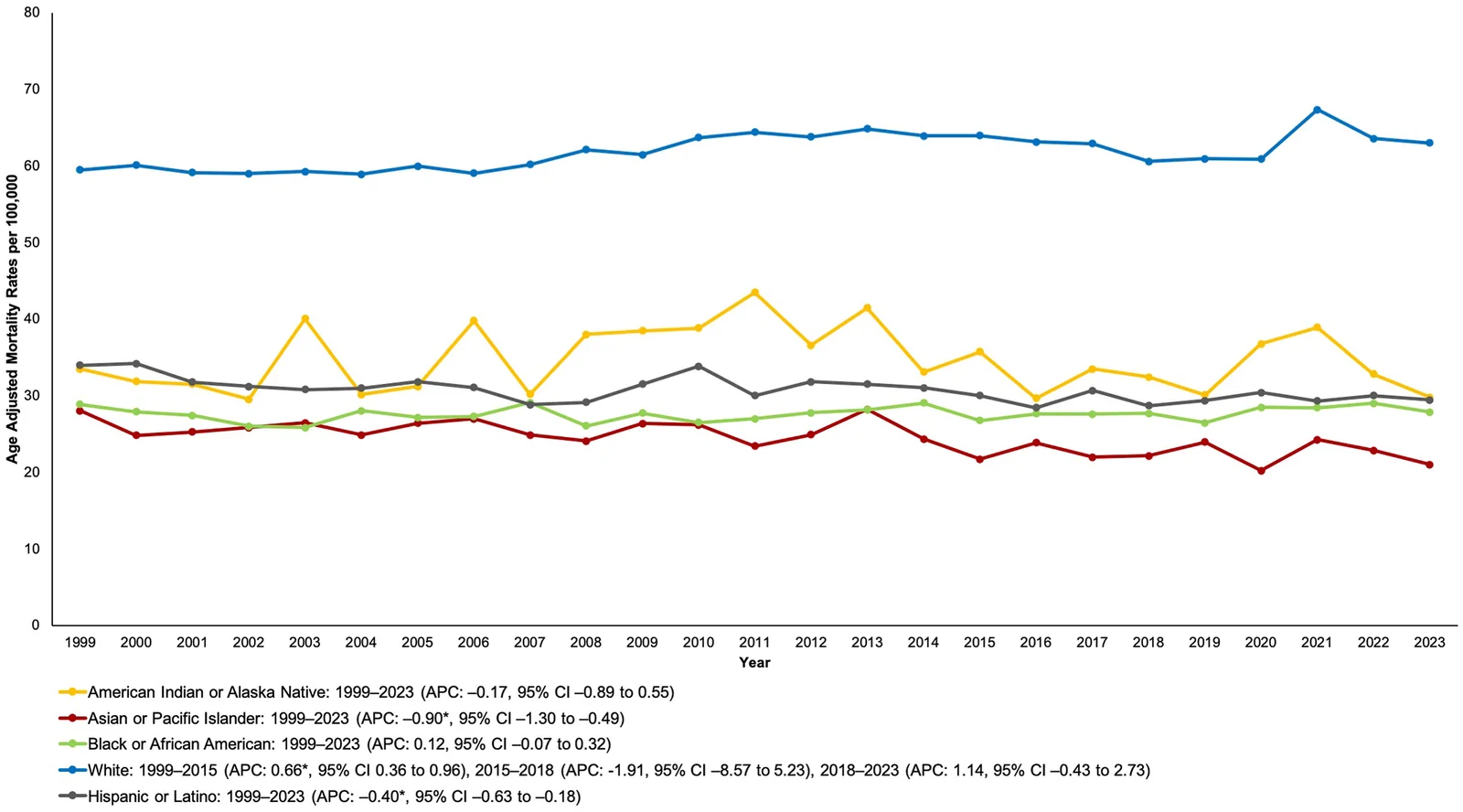
**Trends in age-adjusted aortic stenosis–related mortality rates stratified by race/ethnicity among adults in the United States, 1999 to 2023.** ∗Indicates that the annual percent change (APC) is significantly different than 0 at α = 0.05

### Mortality trends by region

#### Census regions

Over the course of the study, we observed disparities across regions in the US. In 1999, the American West had the highest AAMR (63.95), followed by the Northeast, Midwest, and South (61.96, 56.51, and 46.64, respectively). However, in 2024, the Midwest had the highest AAMR (60.91), followed by the Northeast, West, and South (57.41, 56.19, and 42.87, respectively). Significant increase in mortality was seen in the Midwest (AAPC, 0.38; *P* < .001); however, a significant decrease in mortality was seen in the West (AAPC, –0.34; *P* = .01), Northeast (AAPC, –0.3; *P* = .03), and South (AAPC, –0.33; *P* <.001) (Supplemental Table S4, Figure 4). A significant increase in mortality followed by a significant decrease was seen for the Western region from 1999 to 2011 (APC, 0.54; 95% CI, 0.06-1.50; *P* = .03) and from 2011 to 2024 (APC, –1.15; 95% CI, –1.80 to –0.77; *P* < .001). A similar trend was seen in the Northeastern region from 2006 to 2011 (APC, 2.53; 95% CI, 0.80-5.40; *P* = .04) and form 2011 to 2024 (APC, –1.01; 95% CI, –1.55 to –0.59; *P* = .02). A significant increase was seen in mortality for the Midwestern region from 2004 to 2015 (APC, 1.46; 95% CI, 1.25-2.31; *P* <.001). A decline in mortality was noted from 2015 to 2018 (APC, –1.69; 95% CI, –2.66 to –0.31; *P* = .02) followed by an increase from 2018 to 2021 (APC, 2.94; 95% CI, 1.41-4.04; *P* < .001), and a subsequent decline from 2021 to 2024 (APC, –2.50; 95% CI, –4.30 to –1.54; *P* < .001). A significant decrease was seen in mortality for the Southern region from 1999 to 2024 (APC, –0.33; 95% CI, –0.48 to –0.17; *P* < .001).

**Figure 4:**
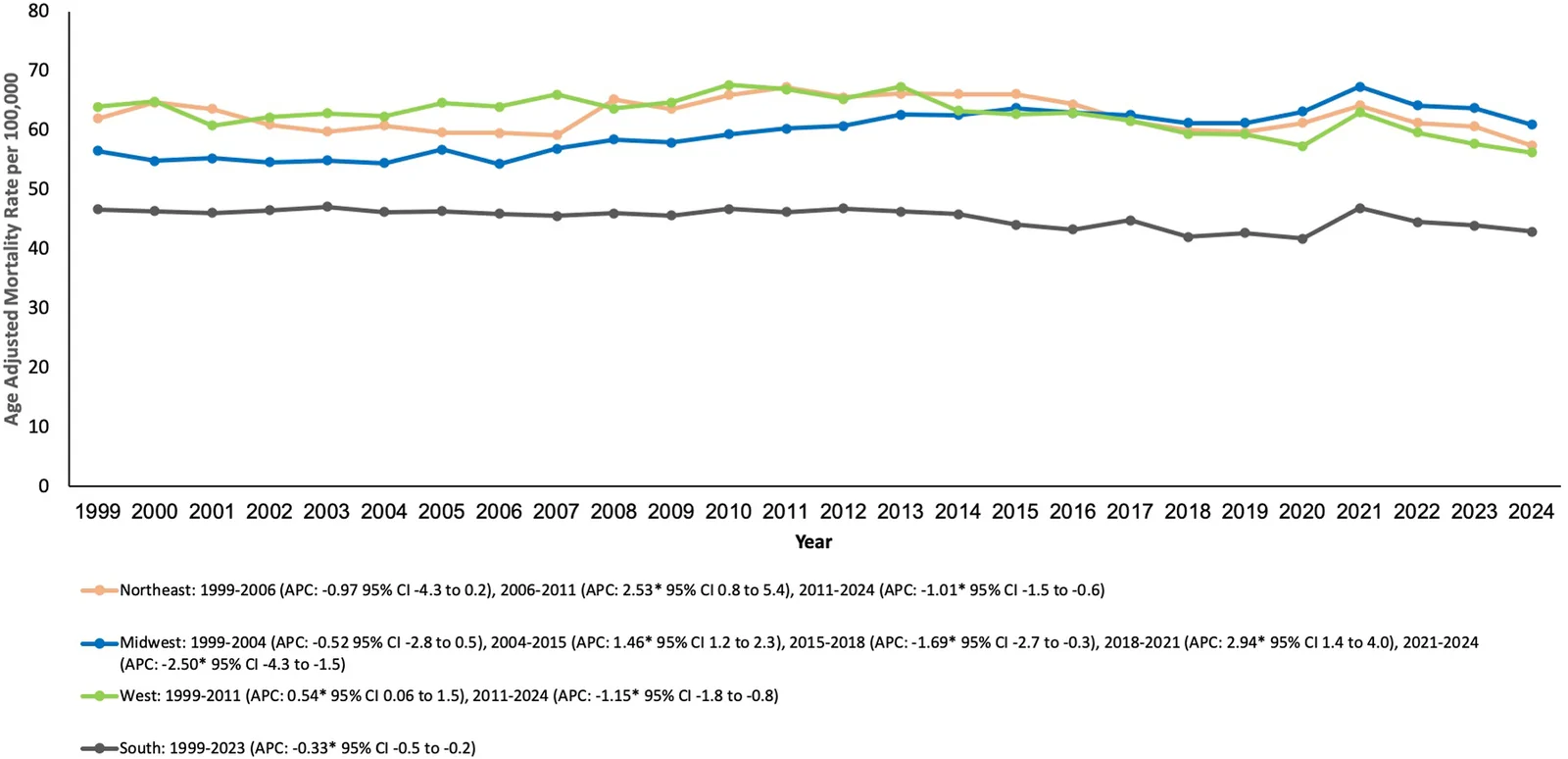
**Trends in age-adjusted aortic stenosis–related mortality rates stratified by geographic region among adults in the United States, 1999 to 2020.** ∗Indicates that the annual percent change (APC) is significantly different than 0 at α = 0.05.

#### Urban-rural

Between 1999 and 2020, large (AAPC, –0.58; *P* < .001) (overall AAMR, 51.96) and medium metropolitan areas (AAPC, –0.02; *P* = .88) (overall AAMR, 59.63) reported a decreasing trend compared to small metropolitan (AAPC, 0.13; *P* = .48) (overall AAMR, 62.09) and nonmetropolitan areas (AAPC, 0.49; *P* = .02) (overall AAMR, 59.64) reporting an increase in mortality. A significant decrease was seen in mortality in the large metropolitan areas from 1999 to 2004 (APC, –1.00; 95% CI, –1.97 to –0.01; *P* = .047) followed by a significant increase from 2004 to 2013 (APC, 0.70; 95% CI, 0.25-1.15; *P* = .005) and another decline from 2013 to 2020 (APC, –1.91; 95% CI, –2.41 to –1.41; *P* < .001). Mortality for medium metropolitan areas showed an increase from 1999 to 2013 (APC, 0.49; 95% CI, 0.23-0.76; *P* < .001), followed by a decrease from 2013 to 2020 (APC, –1.04; 95% CI, –1.69 to –0.39; *P* = .004). A similar trend was seen in the small metropolitan areas with mortality increasing from 1999 to 2014 (APC, 0.54; 95% CI, 0.23-0.86; *P* = .002), followed by a decline from 2014 to 2020 (APC, –0.89; 95% CI, –1.99 to 0.23; *P* = .11). Mortality for nonmetropolitan areas increased from 1999 to 2006 (APC, 0.46; 95% CI, 0.09-0.84; *P* = .02) and 2006 to 2015 (APC, 1.26; 95% CI, 0.97-1.54; *P* < .001), followed by a decline from 2015 to 2018 (APC, –3.00; 95% CI, –5.38 to –0.56; *P* =.02) and a subsequent increase from 2018 to 2020 (APC, 2.48; 95% CI, 0.03-4.98; *P* = .04) (Supplemental Table S5).

#### State

Age-adjusted mortality rates varied greatly among the states, from 124.80 in Vermont to 32.85 in Alabama. States in the 90th percentile included Maine, New Hampshire, Washington, Oregon, and Vermont. On the other hand, states in the bottom 10th percentile included Alabama, Mississippi, Georgia, New Mexico, and Arkansas (Supplemental Table S6).

## Discussion

Our analysis of national mortality data shows that AS-related mortality among older adults in the US has declined over the past 2 decades, although temporal variability was observed. This overall trajectory likely reflects evolving management of severe AS and expanding access to definitive valve therapies. Although historically considered a degenerative process, AS is now recognized as an active disease involving inflammatory and calcific pathways within the valve.^11^ Pharmacologic strategies have not shown meaningful effects on disease progression, making valve replacement the only effective therapy for severe AS.^12^ Early aortic valve replacement has been associated with reduced mortality and cardiovascular hospitalizations,^13^ and the rapid expansion of TAVR has substantially broadened treatment eligibility and improved procedural outcomes.^14^^,^^15^ Although causality cannot be established, the observed decline in AS-related mortality coincided with broader adoption of transcatheter valve therapies across progressively lower surgical risk populations.

Our findings are broadly consistent with a prior national analysis using the CDC WONDER data from 1999 to 2020, which reported a modest decline in AS-related mortality after an initial rise until 2014.^6^ Although the overall trajectory in our analysis is similar, our study extends these observations through 2024, thereby providing additional insight into more contemporary mortality patterns in the era of widespread TAVR adoption. Contemporary national registry and inpatient data have demonstrated increasing TAVR utilization alongside declining SAVR volumes, providing important temporal context for the mortality trends observed in our study. However, CDC WONDER does not include procedure-level data, and therefore, our analysis cannot directly link individual-level valve intervention, TAVR/SAVR utilization, or treatment access to mortality outcomes. Importantly, our study focused only on older adults with nonrheumatic AS, whereas the inclusion of rheumatic AS in the prior study may have confounded their findings because of the differing pathophysiological mechanisms in both etiologies.^6^ In addition, our analysis restricted mortality rate calculations to individuals aged 65 years and above—the population most affected by AS—whereas the prior study used the entire adult population as the denominator, which may have diluted disease-specific mortality estimates.^6^ Furthermore, our analysis incorporated more granular age stratification and urban-rural classifications, enabling identification of important disparities such as increasing mortality among individuals aged ≥85 years and higher mortality in nonmetropolitan populations.

Our age-stratified analysis demonstrated progressively increasing mortality with advancing age. AS prevalence increases substantially with aging, affecting approximately 2% to 3% of individuals older than 65 years and up to 7% of those older than 80 years.^16^ Consistent with prior population studies, this pattern likely reflects cumulative exposure to risk factors, progressive calcific degeneration of the valve, and increasing comorbidity burden with aging. Stewart et al^17^ reported aortic valve sclerosis in 20% of individuals aged 65 to 74 years, increasing to 35% in those aged 75 to 84 years, and 48% in those older than 85 years. In alignment with this epidemiology, individuals aged ≥85 years exhibited the highest mortality rates in our study, whereas modest reductions were observed among individuals aged 75 to 84 years. These findings highlight the persistent mortality burden among the oldest age groups. Nonetheless, the overall improvements across different age groups may reflect, in part, broader changes in AS diagnosis, referral patterns, and improved access to valve replacement therapies, including TAVR; however, this interpretation remains hypothesis-generating given the absence of patient-level treatment data in CDC WONDER.^18^

Sex-based differences in mortality were also observed, with men demonstrating consistently higher mortality throughout the study period. Although mortality declined modestly among women, trends among men remained relatively stable. These differences may partly reflect the higher prevalence of cardiovascular risk factors among men, including coronary artery disease, diabetes, hypertension, and smoking.^19^ Sex-related differences in disease recognition and management of VHD have also been described, with women remaining historically underrepresented in pivotal clinical trials informing guideline recommendations.^20^^,^^21^

Racial disparities were also evident in our analysis. Mortality remained the highest among NH White individuals, whereas NH Asian or Pacific Islander populations exhibited the lowest mortality rates. Differences in health care access, referral patterns, and utilization of valve replacement therapies may contribute to these patterns. Prior studies have reported lower rates of both SAVR and TAVR among Black patients compared with White patients, which may reflect disparities in diagnosis, referral, and access to specialized cardiovascular care rather than true differences in disease burden.^22^^,^^23^ Similarly, lower prevalence and severity of AS reported among Asian populations may partly explain the lower mortality observed in this subgroup.^24^^,^^25^ These patterns are consistent with prior analyses examining racial disparities in the management of AS and may also reflect broader systemic factors, including patient-provider communication and health care access.^26^, ^27^, ^28^

We observed notable geographic differences in AS-related mortality across US regions, levels of urbanization, and individual states. Higher mortality observed in nonmetropolitan regions and increasing mortality trends in the Midwest may reflect disparities in access to specialized cardiovascular services, including structural heart programs capable of performing valve interventions. Prior work has demonstrated that limited access to echocardiography, delayed referral, and longer travel distances to tertiary centers can adversely affect outcomes in patients with severe AS.^29^, ^30^, ^31^, ^32^, ^33^, ^34^ State-level variation further suggests that regional health care infrastructure, referral networks, and practice patterns influence access to timely valve replacement therapies, consistent with prior studies demonstrating geographic variation in the utilization and outcomes of TAVR across the US.^35^

Targeted strategies may help address these disparities. Telemedicine and remote technologies may facilitate heart team collaboration and procedural planning for patients in geographically remote areas. Artificial intelligence–based screening and risk stratification tools may enable earlier diagnoses and more efficient referral pathways for valve intervention. Optimizing procedural workflows and expanding structural heart programs could further increase procedural capacity, allowing more patients to receive timely intervention.

This study has several limitations. Mortality estimates derived from death certificate data may be subject to misclassification or underreporting of AS diagnoses. Prior work has reported a sensitivity of approximately 53% for identifying AS using administrative claims, suggesting potential undercoding of clinically confirmed disease.^36^ In addition, increased utilization and accessibility of echocardiography over the study period may have improved detection and diagnostic ascertainment of AS, potentially contributing to ascertainment bias and influencing observed temporal mortality trends. Furthermore, the database captures deaths among individuals with AS rather than deaths directly attributable to AS. The database also lacks detailed clinical information regarding disease severity, treatment strategies, and comorbid conditions that may influence mortality outcomes. Additionally, although national TAVR/SAVR utilization trends provide temporal context, our data set does not permit direct assessment of whether changes in procedural access or treatment utilization caused the observed mortality trends. Finally, mortality analyses based on race, urbanization, and state-level trends were limited to data available through 2020 because of incomplete or inconsistent reporting of these variables in more recent data sets. A brief fluctuation in mortality during the late study period may also reflect broader health care disruptions during the coronavirus disease 2019 pandemic.

## Conclusion

In conclusion, AS-related mortality in the US declined modestly between 1999 and 2024. However, substantial disparities persisted across demographic and geographic subgroups, with higher mortality observed among men, older adults, specifically those aged ≥85 years, and residents of nonmetropolitan regions. Racial differences in mortality patterns were also evident. These findings underscore the need for improved access to timely diagnosis and advanced valve therapies to ensure that advances in AS management translate into equitable improvements in outcomes among older adults.

## CRediT authorship contribution statement

**Aymen Ahmed:** Writing – review & editing, Writing – original draft, Visualization, Validation, Supervision, Software, Methodology, Investigation, Formal analysis, Data curation, Conceptualization. **Muteia Shakoor:** Writing – original draft, Visualization, Formal analysis, Data curation. **Usama Hussain Kamal:** Writing – original draft, Software, Resources, Methodology, Formal analysis, Data curation. **Hiba Sohail:** Writing – original draft, Visualization, Investigation, Formal analysis, Data curation. **Muhammad Hasnain Mankani:** Writing – original draft, Software, Resources, Methodology, Data curation. **Vikash Jaiswal:** Writing – original draft, Software, Resources. **Najam Us Saqib:** Writing – review & editing, Visualization, Validation, Supervision, Methodology. **Nso Nso:** Writing – review & editing, Visualization, Validation, Supervision, Methodology, Formal analysis. **Arman Qamar:** Writing – review & editing, Validation, Supervision. **Mark J. Ricciardi:** Writing – review & editing, Validation, Supervision.

## Declaration of competing interest

The authors declared no potential conflicts of interest with respect to the research, authorship, and/or publication of this article.

## Footnotes

To access the supplementary material accompanying this article, visit the online version of the *Journal of the Society for Cardiovascular Angiography & Interventions* at 10.1016/j.jscai.2026.105573.
