# Supplementary material for: Trends and Disparities in Aortic Stenosis–Related Mortality Among Adults Aged ≥65 Years in the United States From 1999 to 2024
Source: J Soc Cardiovasc Angiogr Interv. 2026 Aug 11;5(10):105573. doi: 10.1016/j.jscai.2026.105573 (PMC13700030; doi:10.1016/j.jscai.2026.105573)
Supplement: Supplementary file 1 [file mmc1.docx]

**Supplementary Material**

**Supplemental Table S1.** Overall and sex-stratified AS-related age-adjusted mortality rates per 100,000 among adults in the United States, 1999 to 2024

**Supplemental Table S2.** AS-related crude mortality rates per 100,000 among adults stratified by 5-year age groups in the United States, 1999-2024.

**Supplemental Table S3.** AS-related age-adjusted mortality rates per 100,000 among adults stratified by race/ethnicity in the United States, 1999 to 2023.

**Supplemental Table S4**. AS related age-adjusted mortality rates per 100,000 among adults in the United States, 1999-2024.

**Supplemental Table S5.** AS-related age adjusted mortality rates per 100,000 among adults stratified by urban-rural classification in the United States, 1999 to 2020.

**Supplemental Table S6.** AS-related age adjusted mortality rates per 100,000 among adults stratified by states in the United States, 1999 to 2020.

**Supplemental Table S1.** Overall and sex-stratified AS-related age-adjusted mortality rates per 100,000 among adults in the United States, 1999 to 2024

| **Year** | **Overall** | **Male** | **Female** |
| --- | --- | --- | --- |
| 1999 | 55.74 | 64.11 | 50.67 |
| 2000 | 55.99 | 63.79 | 51.43 |
| 2001 | 55.06 | 62.54 | 50.49 |
| 2002 | 54.76 | 62.56 | 50.01 |
| 2003 | 54.95 | 62.73 | 50.1 |
| 2004 | 54.62 | 62.69 | 49.84 |
| 2005 | 55.45 | 62.42 | 51.23 |
| 2006 | 54.61 | 62.01 | 49.98 |
| 2007 | 55.42 | 63.86 | 50.27 |
| 2008 | 56.7 | 65.5 | 51.36 |
| 2009 | 56.3 | 65.21 | 50.74 |
| 2010 | 58.15 | 68.07 | 51.91 |
| 2011 | 58.28 | 67.4 | 52.5 |
| 2012 | 57.82 | 68.94 | 50.91 |
| 2013 | 58.59 | 68.79 | 52.14 |
| 2014 | 57.54 | 67.77 | 50.89 |
| 2015 | 57.02 | 66.77 | 50.52 |
| 2016 | 56.21 | 65.99 | 49.71 |
| 2017 | 55.84 | 65.02 | 49.66 |
| 2018 | 53.66 | 62.68 | 47.65 |
| 2019 | 53.78 | 62.85 | 47.54 |
| 2020 | 53.68 | 64.27 | 46.24 |
| 2021 | 58.28 | 70.09 | 49.94 |
| 2022 | 55.41 | 67.33 | 47.3 |
| 2023 | 54.49 | 65.07 | 47.06 |
| 2024 | 52.46 | 62.34 | 45.41 |

**Supplemental Table S2.** AS-related crude mortality rates per 100,000 among adults stratified by 5-year age groups in the United States, 1999-2024.

| **Year** | **65-69 years** | **70-74 years** | **75-79 years** | **80-84 years** | **85+ years** |
| --- | --- | --- | --- | --- | --- |
| 1999 | 7.12 | 14.80 | 35.15 | 86.62 | 247.21 |
| 2000 | 7.21 | 15.53 | 35.17 | 81.85 | 253.04 |
| 2001 | 7.04 | 15.37 | 33.63 | 81.08 | 248.56 |
| 2002 | 6.16 | 14.46 | 32.66 | 78.72 | 253.48 |
| 2003 | 6.45 | 14.83 | 32.02 | 77.76 | 255.50 |
| 2004 | 6.13 | 14.13 | 31.84 | 75.64 | 257.40 |
| 2005 | 5.90 | 13.62 | 31.29 | 75.87 | 266.10 |
| 2006 | 6.45 | 13.80 | 30.46 | 74.73 | 259.89 |
| 2007 | 5.68 | 13.60 | 30.96 | 74.92 | 267.64 |
| 2008 | 6.12 | 13.52 | 31.73 | 75.37 | 275.32 |
| 2009 | 6.09 | 14.09 | 32.94 | 76.71 | 267.36 |
| 2010 | 6.10 | 14.05 | 32.84 | 77.52 | 281.63 |
| 2011 | 6.29 | 13.78 | 31.30 | 79.48 | 282.74 |
| 2012 | 6.20 | 12.78 | 31.26 | 79.66 | 282.15 |
| 2013 | 6.48 | 13.49 | 31.86 | 79.33 | 286.49 |
| 2014 | 6.11 | 13.72 | 31.95 | 77.56 | 281.34 |
| 2015 | 6.63 | 14.09 | 31.40 | 75.80 | 279.09 |
| 2016 | 6.44 | 13.64 | 30.47 | 72.20 | 280.13 |
| 2017 | 6.66 | 14.19 | 30.50 | 71.33 | 276.69 |
| 2018 | 6.35 | 14.02 | 28.73 | 68.61 | 267.03 |
| 2019 | 6.38 | 14.21 | 30.54 | 68.70 | 264.30 |
| 2020 | 6.68 | 14.70 | 31.26 | 68.14 | 261.22 |
| 2021 | 7.14 | 15.22 | 34.33 | 71.45 | 287.88 |
| 2022 | 7.34 | 15.22 | 33.00 | 71.70 | 267.18 |
| 2023 | 6.93 | 14.23 | 30.96 | 67.34 | 271.11 |
| 2024 | 6.87 | 14.60 | 30.56 | 67.94 | 254.43 |

**Supplemental Table S3.** AS-related age-adjusted mortality rates per 100,000 among adults stratified by race/ethnicity in the United States, 1999 to 2023.

| **Year** | **NH White** | **NH Black or African American** | **NH Asian or Pacific Islander** | **NH American Indian or Alaskan Native** | **Hispanic or Latino** |
| --- | --- | --- | --- | --- | --- |
| 1999 | 59.51 | 28.9 | 28.07 | 33.51 | 34.02 |
| 2000 | 60.1 | 27.96 | 24.89 | 31.9 | 34.23 |
| 2001 | 59.16 | 27.47 | 25.33 | 31.56 | 31.81 |
| 2002 | 59.03 | 26.04 | 25.9 | 29.56 | 31.26 |
| 2003 | 59.30 | 25.87 | 26.51 | 40.08 | 30.85 |
| 2004 | 58.93 | 28.06 | 24.93 | 30.19 | 31.01 |
| 2005 | 59.98 | 27.19 | 26.46 | 31.23 | 31.85 |
| 2006 | 59.05 | 27.32 | 27.01 | 39.83 | 31.12 |
| 2007 | 60.19 | 29.13 | 24.91 | 30.25 | 28.85 |
| 2008 | 62.13 | 26.1 | 24.11 | 38 | 29.17 |
| 2009 | 61.46 | 27.77 | 26.42 | 38.5 | 31.53 |
| 2010 | 63.7 | 26.55 | 26.25 | 38.84 | 33.88 |
| 2011 | 64.42 | 27.04 | 23.48 | 43.52 | 30.05 |
| 2012 | 63.81 | 27.82 | 24.97 | 36.6 | 31.84 |
| 2013 | 64.88 | 28.21 | 28.27 | 41.49 | 31.56 |
| 2014 | 63.95 | 29.09 | 24.39 | 33.15 | 31.07 |
| 2015 | 64.00 | 26.79 | 21.75 | 35.77 | 30.06 |
| 2016 | 63.15 | 27.68 | 23.9 | 29.7 | 28.47 |
| 2017 | 62.91 | 27.64 | 22.02 | 33.51 | 30.7 |
| 2018 | 60.61 | 27.72 | 22.2 | 32.46 | 28.73 |
| 2019 | 60.94 | 26.51 | 23.98 | 30.16 | 29.41 |
| 2020 | 60.91 | 28.5 | 20.25 | 36.77 | 30.44 |
| 2021 | 67.37 | 28.48 | 24.29 | 38.93 | 29.36 |
| 2022 | 63.60 | 29.05 | 22.87 | 32.87 | 30.05 |
| 2023 | 63.03 | 27.9 | 21.04 | 29.85 | 29.47 |

**Supplemental Table S4**. AS related age-adjusted mortality rates per 100,000 among adults in the United States, 1999-2024.

| **Year** | **Northeast** | **Midwest** | **South** | **West** |
| --- | --- | --- | --- | --- |
| 1999 | 61.96 | 56.51 | 46.64 | 63.95 |
| 2000 | 64.67 | 54.85 | 46.39 | 64.79 |
| 2001 | 63.61 | 55.25 | 46.07 | 60.81 |
| 2002 | 60.96 | 54.61 | 46.51 | 62.17 |
| 2003 | 59.77 | 54.88 | 47.11 | 62.87 |
| 2004 | 60.77 | 54.45 | 46.21 | 62.32 |
| 2005 | 59.63 | 56.72 | 46.36 | 64.57 |
| 2006 | 59.52 | 54.33 | 45.91 | 63.96 |
| 2007 | 59.17 | 56.85 | 45.54 | 66.02 |
| 2008 | 65.18 | 58.39 | 46.00 | 63.67 |
| 2009 | 63.61 | 57.90 | 45.65 | 64.71 |
| 2010 | 65.94 | 59.29 | 46.73 | 67.63 |
| 2011 | 67.27 | 60.28 | 46.18 | 66.89 |
| 2012 | 65.53 | 60.70 | 46.83 | 65.24 |
| 2013 | 66.17 | 62.61 | 46.29 | 67.30 |
| 2014 | 66.09 | 62.54 | 45.85 | 63.28 |
| 2015 | 66.08 | 63.70 | 44.09 | 62.66 |
| 2016 | 64.38 | 62.94 | 43.24 | 62.88 |
| 2017 | 61.54 | 62.58 | 44.79 | 61.59 |
| 2018 | 59.95 | 61.21 | 42.01 | 59.41 |
| 2019 | 59.67 | 61.24 | 42.69 | 59.30 |
| 2020 | 61.22 | 63.10 | 41.75 | 57.33 |
| 2021 | 64.16 | 67.35 | 46.86 | 62.97 |
| 2022 | 61.22 | 64.20 | 44.51 | 59.61 |
| 2023 | 60.60 | 63.71 | 43.96 | 57.70 |
| 2024 | 57.41 | 60.91 | 42.87 | 56.19 |

**Supplemental Table S5.** AS-related age adjusted mortality rates per 100,000 among adults stratified by urban-rural classification in the United States, 1999 to 2020.

| **Year** | **Nonmetropolitan** | **Small Metropolitan** | **Medium Metropolitan** | **Large Metropolitan** |
| --- | --- | --- | --- | --- |
| 1999 | 55 | 59.77 | 58.99 | 53.97 |
| 2000 | 56.57 | 61.42 | 59.65 | 53.24 |
| 2001 | 56.5 | 58.23 | 57.49 | 52.91 |
| 2002 | 56.65 | 59.59 | 58.23 | 51.62 |
| 2003 | 56.31 | 62.86 | 57.8 | 51.64 |
| 2004 | 57.08 | 59.54 | 57.68 | 51.47 |
| 2005 | 57.13 | 63.19 | 59.97 | 51.44 |
| 2006 | 56.22 | 60.7 | 57.54 | 51.5 |
| 2007 | 58.43 | 60.15 | 58.8 | 51.93 |
| 2008 | 59.2 | 60.29 | 60.78 | 53.41 |
| 2009 | 60.85 | 61.19 | 60.34 | 52.04 |
| 2010 | 60.44 | 64.02 | 62.01 | 54.49 |
| 2011 | 62.05 | 63.63 | 61.39 | 54.58 |
| 2012 | 61.47 | 64.47 | 62.04 | 53.6 |
| 2013 | 62.89 | 64.56 | 61.53 | 54.77 |
| 2014 | 63.47 | 64.67 | 60.14 | 53.01 |
| 2015 | 64.81 | 63.59 | 61.02 | 51.49 |
| 2016 | 61.29 | 61.61 | 59.85 | 51.93 |
| 2017 | 60.13 | 64.96 | 60.53 | 50.65 |
| 2018 | 59.4 | 61.57 | 58.15 | 48.36 |
| 2019 | 60.2 | 60.5 | 57.19 | 48.94 |
| 2020 | 61.7 | 61.45 | 57.49 | 47.94 |

**Supplemental Table S6.** AS-related age adjusted mortality rates per 100,000 among adults stratified by states in the United States, 1999 to 2020.

| **State** | **Age Adjusted Rate** |
| --- | --- |
| Alabama | 32.85 |
| Mississippi | 33.16 |
| Georgia | 34.24 |
| New Mexico | 35.63 |
| Arkansas | 36.21 |
| District of Columbia | 37.27 |
| Kentucky | 39.11 |
| Louisiana | 39.59 |
| Arizona | 40.62 |
| Oklahoma | 40.95 |
| Nevada | 41.56 |
| Utah | 42.25 |
| Florida | 43.77 |
| Texas | 44.22 |
| New York | 46.04 |
| Illinois | 48.24 |
| Colorado | 48.82 |
| Tennessee | 49.87 |
| West Virginia | 50.34 |
| Virginia | 51.86 |
| Hawaii | 52.39 |
| South Dakota | 52.53 |
| Wyoming | 53.43 |
| Michigan | 53.65 |
| North Dakota | 54.12 |
| South Carolina | 54.15 |
| Kansas | 54.94 |
| Missouri | 54.96 |
| Connecticut | 55.29 |
| North Carolina | 55.68 |
| Indiana | 57.22 |
| Ohio | 59.19 |
| Rhode Island | 59.95 |
| Maryland | 60.05 |
| California | 61.4 |
| Montana | 62.79 |
| Massachusetts | 65.15 |
| Nebraska | 65.19 |
| New Jersey | 65.38 |
| Delaware | 68.39 |
| Iowa | 69.57 |
| Wisconsin | 75.23 |
| Alaska | 75.6 |
| Idaho | 75.96 |
| Minnesota | 77.71 |
| Pennsylvania | 78.28 |
| Maine | 84.26 |
| New Hampshire | 86.71 |
| Washington | 94.94 |
| Oregon | 110.75 |
| Vermont | 124.8 |
